# Ammonium transceptors: Novel regulators of fungal development

**DOI:** 10.1371/journal.ppat.1008059

**Published:** 2019-11-07

**Authors:** Bert van den Berg, Siobhan Lister, Julian C. Rutherford

**Affiliations:** Institute for Cell and Molecular Biosciences, Medical School, Newcastle University, Newcastle upon Tyne, United Kingdom; Geisel School of Medicine at Dartmouth, UNITED STATES

## Introduction

Transceptors are nutrient transporters that act as receptors to regulate downstream signaling pathways. Ammonium transceptors regulate fungal morphology in response to limiting levels of ammonium, which is utilized as a nitrogen source. Few studies have addressed to what extent ammonium transceptors are conserved in pathogenic fungi. Plant and animal fungal pathogens undergo a variety of developmental transitions during infection, and work on some plant fungal pathogens establishes the importance of ammonium transporters during host infection [1, 2]. More widespread studies are needed to determine if ammonium transceptors have a general role in regulating the virulence of fungal pathogens.

## Ammonium transceptors

Fungal ammonium transceptors belong to a large family of ammonium transporters that are found throughout biology, including the human Rhesus proteins [3]. Individual fungal species contain more than one member of this protein family, and in the model fungi that have been studied to date, only one of these acts as an ammonium sensor. In response to limiting ammonium, transceptors regulate morphological change in the yeasts *Saccharomyces cerevisiae* (ScMep2), *Candida albicans* (CaMep2), *Ustilago maydis* (Ump2), and *Cryptococcus neoformans* (Amt2) [4–7]. Ammonium transporters from filamentous fungi also act as transceptors when expressed in *S*. *cerevisiae*, suggesting that this form of signal transduction may be conserved in divergent fungi [8, 9]. Of importance to the classification of these proteins as nutrient receptors, amino acid substitutions have been identified that separate their transport and signaling functions, establishing that this form of ammonium sensing does not relate to nitrogen assimilation but involves some aspect of ammonium conductance through the transceptor [10–13]. Although fungal ammonium transporters are readily identified through homology searches, it is not possible to predict which of these are potential receptors as it is not yet clear what differentiates a transporter that only imports ammonium from one that also senses ammonium availability. Identifying transceptors therefore requires the phenotypic analysis of fungal mutants that lack individual ammonium transporters.

## The structure of the Mep2 transceptor

The high-resolution structures of several ammonium transporters have been determined [14–19]. The basic architecture of these proteins is similar, as they all form stable trimers with each monomer containing a channel through which ammonium is transported. Each monomer contains an extracellular ammonium binding site, a pair of conserved phenylalanine residues that gate the channel, and a twin-histidine motif lining the narrow hydrophobic pore. While the bacterial and human proteins have open conformations, the yeast transceptors as purified are closed due to changes in the positions of their first and third intracellular loops [19]. The yeast transceptors, therefore, appear to need to undergo significant conformational changes to open the transport channel and allow ammonium import. The closed structures make sense as ammonium is potentially toxic, and its import must be carefully regulated. In bacteria, this is achieved by the regulated binding of a P_II_-like signal transduction protein that inhibits ammonium import via binding to the transporter and blocking the pore [20]. Similarly, ammonium transporters from *S*. *cerevisiae* that are not transceptors are controlled by the binding of a target of rapamycin-regulated protein [21]. It is therefore possible that transceptor-mediated ammonium import may be regulated by a different mechanism that controls conformational changes that open the pore of the transporter. Support for this comes from the position of the transceptor C-terminal cytoplasmic domain (CTD), which makes few contacts with the main body of the protein when compared with the CTD of the bacterial transporters [19]. The transceptor CTD may therefore be important to maintain the protein in its closed conformation.

## The mechanism of ammonium sensing

**Fig 1:**
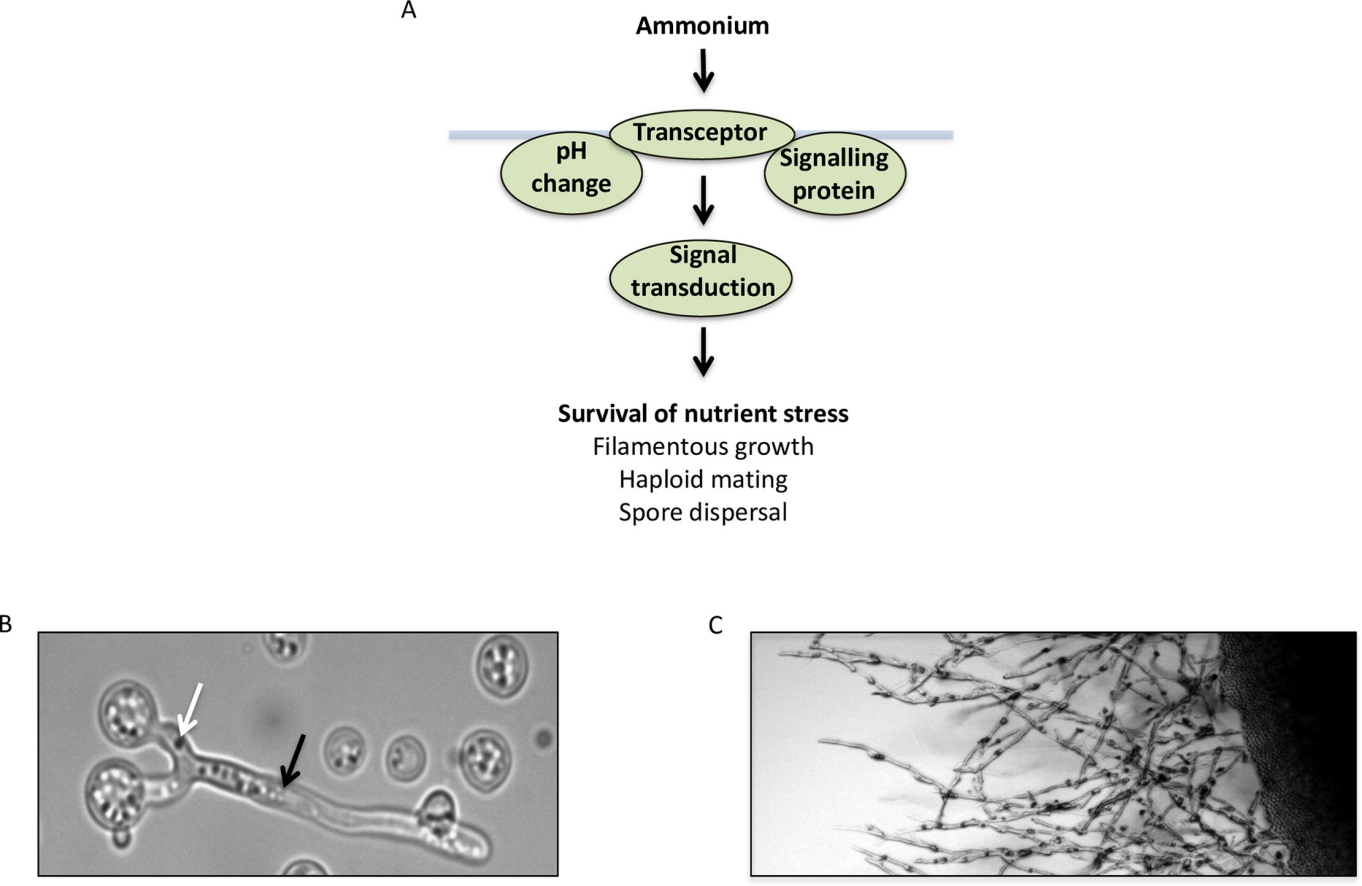
(A) Studies in model dimorphic yeasts support two possible mechanisms of ammonium transceptor function. In the first, ammonium uptake results in cytosolic changes in pH that acts as the signal to initiate morphological change. A different mechanism involves the transceptor physically interacting with a signaling partner to regulate development. The role of the transceptor may be to signal that the levels of ammonium entering the cell are low but sufficient enough to support growth. For saprobic yeasts, this induces processes that promote survival during nutrient stress, such as the induction of filamentous growth. In fungal pathogens, similar mechanisms may regulate morphological changes involved in virulence. (B, C) Low-ammonium conditions can induce mating and filamentous growth. (B) Haploid strains of *Cryptococcus neoformans* mate by producing a conjugation tube (white arrow), followed by the growth of a dikayon filament (black arrow). (C) On low-ammonium agar, haploid strains of *C*. *neoformans* (serotype D) produce filaments that grow away from the main body of the colony. This process is enhanced when the colony is grown near a colony consisting of yeast of the opposite mating type.

**Fig 2:**
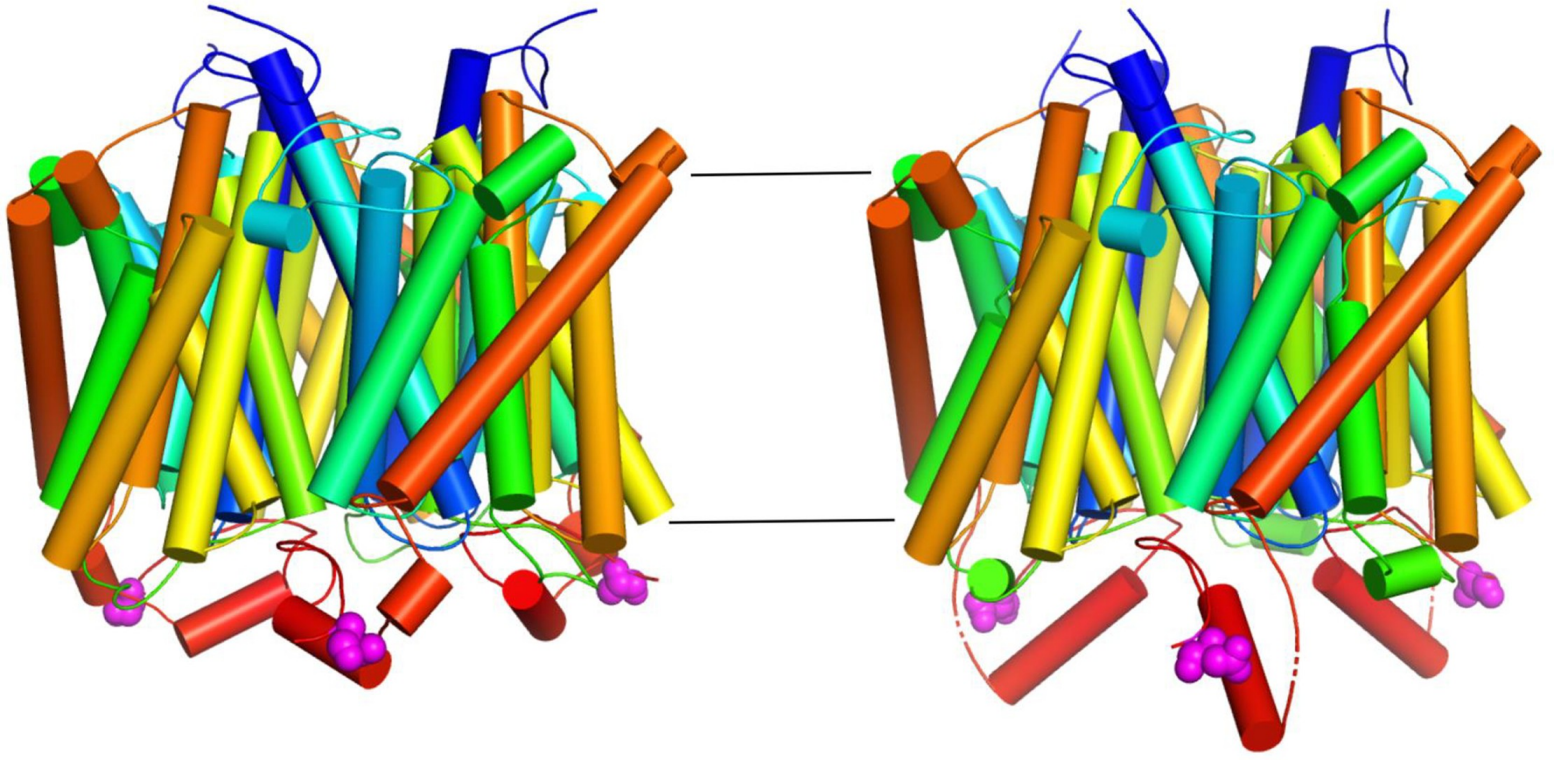
The Mep2 transceptor undergoes a phosphorylation-dependent conformational change. Trimer cartoon models viewed from the side for (left) wild-type *Candida albicans* Mep2 (CaMep2) and (right) a CaMep2 variant that mimics the phosphorylation of a regulatory serine residue (S453) within the cytoplasmic CTD that activates the transporter. The cartoons are in rainbow representation. The phosphorylation-mimicking CaMep2 variant has undergone a large conformational change, resulting in the formation of a 12-residue-long α-helix (red) in the CTD [19]. Changes in transceptor conformation may regulate its interaction with a signaling partner. CTD, C-terminal cytoplasmic domain.

Several questions remain that relate to the mechanisms of both ammonium transport and ammonium signaling by ammonium transceptors. Early studies suggested that ammonium transport by the Amt/Mep/Rh proteins involves the passive diffusion of ammonia gas [15, 16]. Although this likely remains true for the Rhesus proteins, more recent studies on the bacterial and plant transporters are consistent with electrogenic uniport of ammonium ions (NH_4_^+^) or symport of ammonia gas and protons (NH_3_/H^+^), indicating that different mechanisms of ammonium transport are used within this family of proteins [22–28]. Crystal structures and computer simulations of ammonium transport by the *Escherichia coli* AmtB transporter are consistent with the ammonium ion being recruited at an extracellular site. Subsequent stages are less clear, but a plausible scenario is that the ammonium ion passes through the phenylalanine gate and is then deprotonated at a second ammonium binding site [29, 30]. The resulting ammonium gas diffuses through the largely hydrophobic pore, while proton symport most likely occurs via a relay involving the twin-histidine motif [29]. The critical *E*. *coli* AmtB residues for ammonium recruitment and transport are conserved in the ScMep2 and CaMep2 transceptors and occupy identical positions within their ammonium-translocating pore, suggesting a conserved transport mechanism [19]. Remarkably, amino acid substitutions that block ammonium signaling but not ammonium transport by ScMep2 and CaMep2 involve residues that lie within or close to this central pore [10–13]. Presumably, these amino acid substitutions either affect the conformational changes that Mep2 undergoes during ammonium transport or the nature of the transported substrate, in accordance with the two mechanisms that have been proposed to explain the signaling function of ammonium transceptors (Fig 1). The first involves the transceptors acting analogous to G-protein-coupled receptors where the transceptor interacts with a signaling partner that responds to conformational changes in the transporter to regulate a downstream signal transduction pathway [4, 31, 32]. Consistent with this model, the Mep2 CTD undergoes large conformational changes in response to the phosphorylation of a regulatory serine residue within the CTD that activates the transporter [19] (Fig 2). An alternative signaling model proposes that ammonium transport causes changes in cytosolic pH that is then sensed by an internal pH-responsive mechanism [11]. Indeed, a link between pH and polarized growth has been identified in various fungal systems [33–37]. Moreover, a global screen has identified subunits of the vacuolar H^+^-ATPase as being essential for the induction of pseudohyphal growth by *S*. *cerevisiae* [38]. Identifying the mechanism of transport (electrogenic versus electroneutral) by transceptors and their nonsignaling homologues will determine if pH sensing is a potential mechanism that underpins transceptor function.

### Ammonium transceptors and the response to nitrogen starvation

Ammonium transceptors are transcriptionally regulated in response to nitrogen availability so that they are induced when ammonium is absent or at low concentrations within the environment. Transceptor signaling, however, does not occur in the absence of ammonium, as mutants that fail to transport but that are correctly localized to the plasma membrane fail to initiate a dimorphic switch [10, 39]. Therefore, ammonium import when environmental levels of ammonium are low is the signal to induce morphological change. This is consistent with the view that ammonium transceptors regulate processes that promote survival when ammonium levels are limiting but sufficient to support slow growth. These survival strategies involve the switch to filamentous growth by diploid yeast and the mating between compatible haploid yeast, followed by the formation of dikaryon filaments. The diploid form of the baker’s yeast *S*. *cerevisiae* forms chains of elongated cells known as pseudohyphae in response to low-ammonium conditions [40]. Pseudohyphal formation requires a significant change in growth and involves a switch from bipolar to unipolar budding, an increase in apical growth, and the continued adhesion of mother and daughter cells. These pseudohyphal filaments grow away from the area of ammonium limitation, allowing the colony to grow towards new sources of nitrogen [40]. The diploid human pathogenic yeast *C*. *albicans* undergoes a similar transceptor-mediated response by producing branching filaments that grow away from the colony [6]. Although the molecular details of transceptor signaling are not understood, transceptors may regulate the protein kinase A (PKA) or mitogen-activated protein kinase pathways as constitutively active components of these pathways restore the dimorphic switch in transceptor-lacking mutants [4, 6, 10]. Haploid yeast can form filaments to survive limiting nitrogen conditions but will also initiate mating if they are in the presence of a mating partner. Both *C*. *neoformans* and *U*. *maydis* undergo ammonium responsive mating and dikaryon formation, a process that is dependent on an ammonium transceptor [1, 7]. Presumably, the generation of a diploid organism with its potential to produce spores for further dispersal provides an additional strategy to survive nutrient stress.

## Perspectives

Fungal pathogens undergo significant morphological changes that are required for the successful infection of their hosts. These include the dimorphic transition between yeast and filamentous forms and the development of specialized infection structures. The formation and dispersal of spores also allow the spread of infection and can promote genetic variability that results in antifungal resistance. Fungal pathogens can experience nitrogen limitation, and regulatory overlap between pathogenicity and nitrogen starvation genes has been identified, including the induction of ammonium transporter genes during infection [41–45]. Similar to their role in saprobic fungi, ammonium transceptors may therefore regulate morphological change in pathogenic fungi to induce processes that promote their dispersal within or from their hosts. There are a few examples that suggest that this may be the case. A mutant of the broad host range plant pathogen *Colletotrichum gloeosporioides* that lacks the MepB ammonium transporter has reduced levels of PKA activity and appressoria formation and is less virulent than a wild-type strain [2]. Secondary metabolite production by the rice pathogen *Fusarium fuikuroi* is induced in a mutant lacking the MepB ammonium transporter, which may have a transceptor-like role [8]. A more definitive transceptor role during fungal infection has been assigned to the Ump2 ammonium transceptor in the maize pathogen *U*. *maydis*. A mutant lacking Ump2 fails to filament in response to low ammonium, exhibits reduced mating, and is significantly less virulent [1, 5]. Together, these studies support a potential link between ammonium transceptor function and fungal pathogenicity. It is widely accepted that fungal pathogens have a negative impact on human health and agricultural productivity worldwide. Due to increasing pathogen resistance to antifungal agents, there is a timely need for the identification of new antifungal targets. Ammonium transceptors may fall within this category as they support survival during nutrient stress and their accessibility as cell surface proteins makes them ideal antifungal targets. An analogy can be made with human G-protein-coupled receptors that are cell membrane proteins that are the targets of approximately 35% of approved drugs [46]. Further studies are therefore needed to identify the role that ammonium transceptors may have in plant and animal disease and any small molecules that inhibit their signaling function.
